# Coerced Hospital Admission and Symptom Change—A Prospective Observational Multi-Centre Study

**DOI:** 10.1371/journal.pone.0028191

**Published:** 2011-11-30

**Authors:** Thomas W. Kallert, Christina Katsakou, Tomasz Adamowski, Algirdas Dembinskas, Andrea Fiorillo, Lars Kjellin, Anastasia Mastrogianni, Pětr Nawka, Georgi Onchev, Jiri Raboch, Matthias Schützwohl, Zahava Solomon, Francisco Torres-González, Stephen Bremner, Stefan Priebe

**Affiliations:** 1 Klinik für Psychiatrie, Psychosomatik und Psychotherapie, Leipzig, Germany; 2 Unit for Social and Community Psychiatry, Queen Mary, University of London, London, United Kingdom; 3 Department of Psychiatry, Wroclaw Medical University, Wroclaw, Poland; 4 Psychiatric Clinic, Vilnius Mental Health Centre, University of Vilnius, Vilnius, Lithuania; 5 Department of Psychiatry, University of Naples, Naples, Italy; 6 Psychiatric Research Centre, Örebro, Sweden; 7 Psychiatric Hospital, Thessaloniki, Greece; 8 Psychiatric Hospital, Michalovce, Slovak Republic; 9 Department of Psychiatry, Medical University of Sofia, Sofia, Bulgaria; 10 Psychiatric Department, Charles University, Prague, Czech Republic; 11 Department of Psychiatry and Psychotherapy, University of Technology, Dresden, Germany; 12 School of Social Work and Geha Mental Health Center, University of Tel Aviv, Tel Aviv, Israel; 13 Department of Legal Medicine and Psychiatry, Medical Faculty, University of Granada, Granada, Spain; Federal University of Rio de Janeiro, Brazil

## Abstract

**Introduction:**

Coerced admission to psychiatric hospitals, defined by legal status or patient's subjective experience, is common. Evidence on clinical outcomes however is limited. This study aimed to assess symptom change over a three month period following coerced admission and identify patient characteristics associated with outcomes.

**Method:**

At study sites in 11 European countries consecutive legally involuntary patients and patients with a legally voluntary admission who however felt coerced, were recruited and assessed by independent researchers within the first week after admission. Symptoms were assessed on the Brief Psychiatric Rating Scale. Patients were re-assessed after one and three months.

**Results:**

The total sample consisted of 2326 legally coerced patients and 764 patients with a legally voluntary admission who felt coerced. Symptom levels significantly improved over time. In a multivariable analysis, higher baseline symptoms, being unemployed, living alone, repeated hospitalisation, being legally a voluntary patient but feeling coerced, and being initially less satisfied with treatment were all associated with less symptom improvement after one month and, other than initial treatment satisfaction, also after three months. The diagnostic group was not linked with outcomes.

**Discussion:**

On average patients show significant but limited symptom improvements after coerced hospital admission, possibly reflecting the severity of the underlying illnesses. Social factors, but not the psychiatric diagnosis, appear important predictors of outcomes. Legally voluntary patients who feel coerced may have a poorer prognosis than legally involuntary patients and deserve attention in research and clinical practice.

## Introduction

Coerced hospital admissions are commonly practised across the world [1]–[3]. Patients are either legally coerced in line with the given national or regional legislation or their hospital admission is formally voluntary, but they still feel subjectively coerced to accepting the admission. Such coercive measures are widely regarded as an important human rights issue, a position reflected in the UN Convention on the Rights of Persons with Disabilities [4], emphasized by international organizations of patients/(ex-)users and survivors of psychiatry [5], and supported by political bodies such as the Council of Europe [6]. This underlines the particular challenge to provide the best possible treatment for coerced patients, which should be based on sound research evidence.

Systematic research on patients following coerced hospital admission is however limited. Reviews on clinical outcomes of patients [3], [7] suggest that most patients show symptom improvements over time, but also note significant limitations of existing research. Clinical improvement has been commonly assessed on single global functioning scales rather than validated symptom scales, and sample sizes were usually too small to explore predictors of more or less favourable outcomes. Also, previous studies have not considered all coerced patients, i.e. those who are legally coerced and those who are voluntary according to their legal status but feel coerced, in one analysis. Using a more inclusive understanding of coercion appears important since the formal legal status of an admission and patients' subjective experience of coercion often differ [8]. Various studies showed that between 10% and 50% of formally voluntary patients feel in fact coerced to the admission [9]–[11].

Addressing the shortcomings of previous research, the EUNOMIA project studied a large sample of patients following coerced hospital admission at study centres in 11 European countries. Using the same protocol in all countries, we included patients who were legally coerced and those who felt coerced despite a legally voluntary admission [12]. Patients were recruited and assessed within the first week following admission and followed up after one and three months. Taking symptom changes between the first assessment and the two follow ups as outcome criteria, we aimed to identify patient characteristics associated with more or less positive outcomes. A specific aspect was to explore differences in outcomes between three groups of patients defined by their legal status and subjective experience of coercion, i.e. patients who were legally coerced and felt coerced, those who were legally coerced but did not feel coerced, and those who were legally voluntary but felt coerced.

## Methods

We conducted a multicentre prospective cohort study at sites in 11 European countries; i.e. Bulgaria, Czech Republic, Germany, Greece, Italy, Lithuania, Poland, Slovakia, Spain, Sweden, and United Kingdom [13]. Legally voluntary and involuntary inpatients were recruited from acute wards in 1 to 5 hospitals in each country between July 2003 and October 2005. Data on the hospital and other mental health service characteristics in each site have been described in detail elsewhere [12].

The inclusion criteria were: inpatients in general psychiatric departments, between 18 and 65 years, living in the catchment area, sufficient command of the national language, able to give informed consent. Patients were excluded if: they were admitted due to intoxication, had a primary diagnosis of dementia, were transferred from another hospital, or had already taken part in the study at a previous admission [12], [13].

Eligible patients were identified through administrators or staff in the wards upon admission. Once identified, they were approached by researchers (independent from the patients' care) and invited to take part in the study. After complete description of the study to the subjects, written informed consent was obtained. Once written informed consent was received [14], patients were asked to take part in interviews within a week after admission (baseline) and at one and three month follow-ups. All baseline interviews were conducted in the hospital. The follow-up interviews were completed most commonly in the interviewees' homes, and sometimes in the hospital or on the telephone.

We aimed to include all involuntarily admitted patients and those voluntarily admitted patients who felt coerced into admission. Involuntary admissions followed national legislation [15] and routine practice in each country. We attempted to recruit consecutive involuntarily admitted patients. In order to establish whether legally voluntary patients felt coerced, consecutive legally voluntarily admitted patients (or a random selection of voluntarily admitted patients in Germany, Lithuania, Bulgaria and Sweden) were asked to rate their perceived coercion at admission on the McArthur Perceived Coercion Scale (MPCS). The scale measures five dimensions of perceived coercion (i.e. perceived control, choice, influence, freedom and idea) and scores range from 0 to 5 with higher scores indicating higher levels of coercion [12], [16], [17]. Those with a total score of at least 3 on the MPCS were considered coerced and were asked to participate in the study.

The primary outcome was patients' severity of symptoms one month and three months after admission. This was measured with the 24-item version of the Brief Psychiatric Rating Scale (BPRS). The translation of the scale in each national language and the training of all researchers in using the instrument have been described elsewhere [12], [18], [19]. Inter-rater reliability for the BPRS sum score was good (Intraclass correlation coefficient = 0.78).

Baseline socio-demographic and clinical characteristics including patients' initial satisfaction with treatment were tested as potential predictors of outcome [20], [21]. These included: age, gender, employment (i.e. unemployed/pensioned vs. employed), living situation (i.e. living alone vs. living with someone), psychiatric hospitalisation in the past (none vs. at least one), diagnosis according to ICD-10 (collapsed into three groups: schizophrenia or other psychosis, i.e. F20–29; affective disorder, i.e. F30–39; and other F diagnoses), and baseline satisfaction with treatment on the Client's Assessment of Treatment Scale (CAT). This scale comprises seven items and assesses patients' views on whether their treatment is right for them and whether they feel respected, as well as on specific treatment components (i.e. relationships with staff and medication) [22], [23]. Each item is rated from 0 “not at all” to 10 “entirely satisfied” and the mean score was used for further analyses.

Reflecting the legal status and perceived coercion of patients at baseline we then formed three groups: legally involuntary patients with a high level of perceived coercion, i.e. with a total score of at least 3 on the MPCS as described above; legally involuntary patients with a low level of perceived coercion, i.e. with a total score between 0 and 2 on the MPCS; and legally voluntary patients with a high level of perceived coercion.

The study was approved by the relevant Research Ethics committees in each country:

Research Ethics Committee, Medical University Sofia, Sofia, Bulgaria

Ethics committee at the Faculty of Medicine at Dresden University of Technology, Dresden, Germany

Scientific Board of the Psychiatric Hospital of Thessaloniki, Thessaloniki, Greece

IFB Committee of Geha University Hospital and Israeli Ministry of Health, Israel

Ethical Committee of the Second University of Naples, Naples, Italy

Lithuanian Bioethical Committee, Vilnius, Lithuania

Commission of Bioethics at Wroclaw Medical University, Wroclaw, Poland

Ethical Committee (Comité Ético) of University Hospital of San Cecilio. Granada, Spain

Research Ethics Committee of Örebro University Hospital, Örebro, Sweden

East London and The City Research Ethics Committee, London, UK

### Statistical analysis

Descriptive statistics on symptom severity at all time-points and on characteristics potentially associated with symptoms were calculated. We tested for bi-variable associations between all potential predictors and each outcome (i.e. BPRS one and three months after admission), adjusting for BPRS scores at baseline and whether the patient was still in hospital at the time the outcome was assessed. Variables that were significantly associated with outcome in the bi-variable analysis were then entered in a generalised linear model where we also controlled for the above-mentioned variables. Country effects were controlled for by fitting dummy variables. We also fitted interaction terms between country and coercion category to test the hypothesis that, compared to the coerced involuntary patients, non-coerced involuntary and coerced involuntary patients have variable differences in BPRS in the different countries and performed a composite likelihood ratio test of these. All statistical analyses were performed in Stata statistical software version 12.0.

## Results

**Figure 1:**
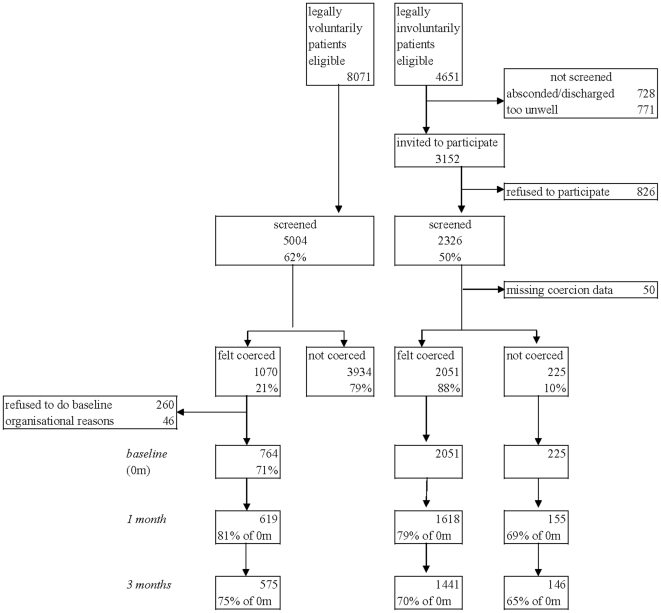
Recruitment and follow-up rates in involuntary and voluntary patients.

Recruitment and follow-up rates for all participants are presented in Figure 1. Overall, 44% of patients were female, 72% of patients were unemployed, 65% lived alone, 72% had at least one previous hospitalisation, and 60% had a diagnosis of schizophrenia (Table 1). The baseline characteristics of patients in the samples that were followed-up at 1 month (and at 3 months) were: 46% (46%) female; 73% (73%) unemployed; 37% (38%) living with others; 73% (74%) with a previous hospitalisation; 63% (62%) diagnosed with schizophrenia; 19% (20%) with affective disorders, and 18% (18%) with ‘other’ diagnoses. The mean age of those followed-up at 1 month was 39.1 (SD = 11.3) and of those followed-up at 3 months 39.3 (SD = 11.2). The mean CAT score in the first week of those followed-up at 1 month was 6.2 (SD = 2.7) and of those followed-up at 3 months 6.2 (SD = 2.7). The mean baseline BPRS score of those followed-up at 1 month was 54.4 (SD = 15.4) and of those followed-up at 3 months 54.6 (SD = 15.5). The characteristics of participants followed-up were similar to those of the whole sample participating in baseline interviews.

**Table 1:** Baseline characteristics of participating patients, severity of symptoms and hospitalisation status at one month and three month follow up.

	Coerced involuntaryN = 2051	Non-coerced involuntaryN = 225	Coerced voluntaryN = 764	Total sampleN = 3090 N (%)
Gender				
Female	910 (44)	73 (33)	364 (48)	1365 (44)
Male	1139 (56)	151 (67)	400 (52)	1722 (56)
Age				
N	2048	223	764	3087
Mean (SD)	38.8 (11.1)	37.8 (11.3)	39.9 (11.6)	39.0 (11.2)
Employment				
No	1484 (73)	142 (64)	544 (72)	2205 (72)
Yes	539 (27)	80 (36)	213 (28)	846 (28)
Living situation				
With others	706 (35)	64 (29)	296 (39)	1074 (35.4)
Alone	1307 (65)	159 (71)	454 (61)	1960 (64.6)
Past hospitalisation				
At least one	1419 (71)	159 (72)	589 (78)	2196 (72.4)
None	586 (29)	63 (28)	169 (22)	837 (27.6)
Diagnosis				
Schizophrenia	1280 (64)	115 (51)	414 (54)	1842 (60.4)
Affective dis.	313 (16)	44 (20)	196 (26)	558 (18.3)
Other	418 (21)	65 (29)	154 (20)	649 (21.2)
Treatment satisfaction (CAT) baseline				
N	1829	217	712	2793
Mean (SD)	5.79 (2.8)	7.4 (2.2)	6.9 (2.4)	6.2 (2.7)
Symptoms (BPRS sum score) baseline				
N	2027	219	758	3053
Mean (SD)	54.0 (15.7)	48.7 (11.9)	52.7 (13.8)	53.2 (14.4)
Symptoms (BPRS sum score) at 1 month				
N	1549	147	604	2335
Mean (SD)	41.7 (12.7)	39.5 (10.6)	41.8 (12.7)	41.6 (12.6)
Symptoms (BPRS sum score) at 3 months				
N	1358	140	510	2035
Mean (SD)	38.0 (11.2)	36.7 (10.1)	38.9 (11.9)	38.1 (11.3)
Still in hospital at 1 month	917 (47)	91 (41)	278 (36)	1302 (43.8)
Still in hospital at 3 months	184 (10)	18 (8)	49 (6)	252 (8.5)

Other than where mean (SD) is stated, figures are n (%).

Symptom severity reduced significantly over time. Comparisons between time points using paired t-tests yielded significant differences in BPRS scores between baseline and 1 month after admission (p<0.001), 1 month and 3 months after admission (p<0.001), and baseline and 3 months after admission (p<0.001).

### Models predicting changes in symptom severity

In the bi-variable models, the largest changes in BPRS at 1 (Table 2) and 3 months (Table 3) were for employment. Compared to the unemployed, the employed scored, on average, 3.1 points less on the BPRS at 1 month, 95% CI 2.2 to 4.1 points less, and 3.7 points less at 3 months (95% CI 2.7 to 4.8). These reductions were diminished in the multivariable model to 2.5 (Table 2) and 3 (Table 3) BPRS points respectively but employment retained significance at the 0.1% level. Compared to the coerced voluntary group, the coerced involuntary group had a BPRS score at 1 month significantly higher by 1.4 points (95% CI 0.4 to 2.5) (Table 2). There was weak evidence of a difference at 3 months: 1.1 points (95% CI −0.1 to 2.2) (Table 3). On adjustment for the other variables, these differences increased slightly. There was no evidence of a difference between the non-coerced and coerced involuntary patients, either at 1 or 3 months (Tables 2 & 3). Overall, coercion was not significantly related to BPRS at 3 months after adjustment for the other variables listed (p-value from likelihood ratio test 0.19). At one and three months, no previous hospitalisation, still being in hospital, and higher treatment satisfaction at baseline were also significantly associated with lower BPRS. The interactions were significant neither at 1 month (p-value = 0.45) nor at 3 months (p-value = 0.26).

**Table 2:** Factors associated with severity of symptoms in bi-variable and multivariable generalised linear models adjusting for country, BPRS baseline scores and whether the patient was still in hospital at 1month.

Predictor variables	Bivariable Models			Multivariable Model^2^		
	*B* 1	95% CI for *B*	*P*-value	*B* 1	95% CI for *B*	*P*-value
Employment	−3.13	−4.08 to −2.17	<0.001	−2.51	−3.53 to −1.48	<0.001
employed vs. unemployed						
Living alone	1.38	0.48 to 2.27	0.002	0.86	−0.08 to 1.79	0.07
Yes vs. no						
Past hospitalisation	−3.26	−4.23 to −2.29	<0.001	−2.76	−3.79 to −1.73	<0.001
No vs. Yes						
Coercion category						
Non-coerced involuntary vs. coerced involuntary	0.85	−0.92 to 2.61	0.346	1.24	−0.53 to 3.01	0.17
Coerced voluntary vs. coerced involuntary	1.44	0.42 to 2.46	0.006	1.49	0.44 to 2.53	0.005
CAT score at baseline	−0.36	−0.54 to −0.19	<0.001	−0.37	−0.55 to −0.19	<0.001
BPRS score at baseline				0.39	0.35 to 0.42	<0.001
Still in hospital 1 month after admission						
No vs. Yes				−1.68	−2.70 to −0.66	0.001
Intercept				24.82	21.54 to 28.11	<0.001

1 *B* = regression coefficient.

2 *P*-value from likelihood ratio test of interaction between coercion category and country was 0.45, therefore results reported are from model without interactions.

**Table 3:** Factors associated with severity of symptoms in bi-variable and multivariable generalised linear models adjusting for country, BPRS baseline scores and whether the patient was still in hospital at 3months.

Predictor variables	Bivariable Models			Multivariable Model^2^		
	*B* 1	95% CI for *B*	*P*-value	*B* 1	95% CI for *B*	*P*-value
Employment	−3.71	−4.76 to −2.65	<0.001	−2.99	−4.14 to −1.84	<0.001
employed vs. unemployed						
Living alone	1.76	0.77 to 2.74	<0.001	1.22	0.18 to 2.25	0.021
Yes vs. no						
Past hospitalisation	−2.76	−3.84 to −1.68	<0.001	−1.93	−3.08 to −0.77	0.001
No vs. Yes						
Coercion category						
Non-coerced involuntary vs. coerced involuntary	0.28	−1.61 to 2.17	0.77	0.44	−1.46 to 2.34	0.65
Coerced voluntary vs. coerced involuntary	1.06	−0.08 to 2.20	0.07	1.08	−0.09 to 2.25	0.07
CAT score at baseline	−0.25	−0.44 to −0.05	0.01	−0.21	−0.41 to −0.01	0.04
BPRS score at baseline				0.22	0.18 to 0.25	<0.001
Still in hospital 3 months after admission						
No vs. Yes				−2.60	−4.19 to −1.00	0.001
Intercept				28.04	24.44 to 31.63	<0.001

1 *B* = regression coefficient.

2 *P*-value from likelihood ratio test of interaction between coercion category and country was 0.26, therefore results reported are from model without interactions.

To explore further the association of coercion at baseline and outcomes, we changed the reference groups in post hoc analyses. The adjusted BPRS scores of legally voluntary patients who felt coerced did not differ significantly from those of legally involuntary patients who did not feel coerced, who were the smallest of the three samples. The regression coefficient and 95% CI were 0.25 (−1.67 to 2.16), p-value 0.801 at 1 month and 0.64 (−1.42 to 2.71), p-value 0.542 at 3 months. When we combined the two legally involuntary groups and, in the regression model, compared this combined sample with the legally voluntary group, the legally voluntary sample had a greater BPRS score than the involuntary group at both time points: 1.37 (0.34 to 2.4), p-value 0.009 at 1 month and 1.04 (−0.12 to 2.19), p-value 0.079 at 3 months.

## Discussion

### Main findings

Following coerced hospital admission, patients show significantly improved symptom levels after one month and further improvements after three months. On average the magnitude of the improvement may be seen as moderate, with substantial inter-individual variation. Apart from higher baseline symptoms, being unemployed, living alone, repeated hospitalisation, and being initially less satisfied with treatment were all associated with less symptom improvement after one month and, other than initial treatment satisfaction, also after three months, whilst the diagnostic group was not linked with outcomes. Adjusting for all other factors, patients with a legally voluntary admission who felt coerced still had poorer outcomes at both follow ups. The size of the difference was small, but it was statistically significant after adjusting for all other factors that were linked with outcomes.

### Strengths and limitations

This is the largest prospective study on outcomes of coerced acute admissions of adult general psychiatric patients to psychiatric hospitals ever conducted and the first one to use the same methods across sites in several countries. It included hospitals in eleven European countries with different legislation [24] and practice of these admissions. The study included patients with legal and subjective definitions of coercion. All patients were recruited and interviewed within the first week after admission and assessed face to face by trained researchers using a validated symptom scale for establishing outcomes. The multivariable statistical analysis was adjusted for the influence of potential confounders, and the large sample size provided sufficient statistical power to interpret negative findings, such as the absence of a predictive value of diagnostic categories.

However, the study also has several limitations. The study was purely observational, and the analysis exploratory. Baseline scores were assessed within the first week, but not at the time of admission and symptoms may have already significantly changed between the time of admission and the first assessment. The study recorded outcomes following involuntary hospital admission over a three month period which might be seen as too short to evaluate the long term impact of coerced admissions. Specific treatment characteristics were not considered as potential predictors of outcome. An important shortcoming is the potential selection bias. The recruitment and follow up rates are in line with rates reported in previous studies [3], [7], [25] and may be seen as satisfactory given the challenging nature of the clientele, but the selection is still substantial and the samples cannot be regarded as representative. Only for the sample in the United Kingdom, data exist for the eligible patients who have not been included in the study [13]. The comparison shows no substantial differences in age, gender, educational level, ethnic group and clinical diagnosis between the included and not-included patients. Yet, other characteristics might be more relevant and no such comparison can be made for the other countries. One may assume that any selection bias would have affected the absolute levels of symptoms and symptom change more than the identification of characteristics associated with outcomes. The latter are based on correlations that are usually more robust against selection bias than the mere distribution of outcome data. Finally, only wide diagnostic categories were used and actual treatment in the hospital, other than length of stay, was not considered in the analysis.

### Comparison against the literature

The sample characteristics are similar to those in other studies on coerced treatment [3], [7], [25]. Most patients are male, without employment and live alone. Also, most of them have experienced previous hospitalisations, are diagnosed with having schizophrenia or a related disorder, and have high symptom levels. Symptoms significantly improve over time which is also consistent with findings in previous smaller studies on coercion [3], [7], [26].

Social factors in form of employment and living with someone predict more favourable outcomes which is in line with numerous studies on outcomes of psychiatric treatment in general [21], [27], [28], although it has not been demonstrated before for patients who were legally or subjectively coerced to hospital admission. A history of previous hospitalisations may indicate a remittent or more persistent course of the underlying illness, and therefore predict less favourable symptom improvements [27]. Finally, the initial satisfaction with psychiatric treatment including voluntary and involuntary hospital treatment has been repeatedly shown to predict short and longer term outcomes [29], [30]. In this study, patients' subjective assessment of hospital treatment within the first week after admission predicted symptom change at one month, but not at three months.

Other patient characteristics such as age and gender were not related to outcomes. It may be worth noting that the diagnostic category was not associated with symptom change either. Coerced admission might select a specific group of severely ill patients in whom social factors, but not the clinical diagnosis are relevant for outcomes. Alternatively, the diagnostic categories used in the analysis were too crude to identify differences.

### Coercion and outcomes

Patients with a legally voluntary admission who felt coerced to hospital treatment showed less positive symptom change. In our study, 21% of all screened patients who were formally voluntary expressed a level of subjective coercion to be included in this category. The exact figure may vary depending on the precise cut off point used and the context, but one may conclude from this and other studies [10], [11] that a substantial proportion of patients fall in this category and that the findings are therefore relevant to clinical practice in many in-patient settings.

Previous studies failed to establish a link between perceived coercion and symptom change, but may have lacked the power to detect such association [31], [32]. In our study the result held true when all potential confounders considered in this study were adjusted for. So, it cannot be explained by different socio-demographic characteristics, higher baseline symptoms or different diagnostic categories. One might conclude that the subjective experience of feeling coerced to hospital treatment may have a particularly negative influence on treatment motivation and therapeutic relationships, and thus outcomes, if the coercion is implemented through informal means rather than the more transparent legal procedures [33]. Such an explanation may be consistent with other studies showing the importance of ‘procedural justice’ for patient experiences and attitudes [34].

### Implications

Clinicians may commonly believe to act in the interest of the patient when they try and persuade a reluctant patient to accept hospital admission. Yet, many of those patients will feel coerced despite a formally voluntary admission, and this study shows that they tend to have poorer outcomes than other groups. This may be a reason to consider alternative options which may be a legally involuntary admission or no admission at all. Once patients are admitted on a legally voluntary basis, feelings of coercion should be explored and potentially addressed.

At the same time, one should consider that the difference in outcomes between the two larger groups in this study, i.e. legally voluntary and involuntary patients who all felt coerced, was rather small and statistically significant only in a very large sample. In individual cases, the legal status can only be regarded as one out of various aspects predicting the expected symptom changes of a patient in the future.

The study suggests that in future research on coercion in psychiatry sensitive legal definitions and subjective experience should be considered as criteria to include and group patients. In-depth studies may be required to understand the processes mediating coercion at admission and symptom change [35]. Interventions within and outside hospitals should be developed and tested to improve outcomes for all coerced patients [33], [36], [37].
